# Bilateral Pleural Effusion Revealing a Rare Breast Tumor: A Case Report

**DOI:** 10.7759/cureus.32312

**Published:** 2022-12-08

**Authors:** Ouiame Nabou, Fatima Zahra Baddi, Oumayma Haloui, Mohammed Musallam, Afaf Thouil, Hatim Kouismi

**Affiliations:** 1 Department of Respiratory Diseases, Mohammed VI University Hospital, Faculty of Medicine and Pharmacy, Mohammed First University, Oujda, MAR; 2 Department of Respiratory Diseases, Mohammed VI University Hospital, Oujda, MAR; 3 Research and Medical Sciences Laboratory (LRSM), Faculty of Medicine and Pharmacy, Mohammed First University, Oujda, MAR

**Keywords:** pleurodesis, lobular carcinoma, breast cancer, metastasis, pleural effusion

## Abstract

The most common tumors causing pleural metastasis in women are gynecological cancers, with breast cancer at the top of the list. However, the revelation of the latter by pleural effusion is rare. We report the case of a 61-year-old woman, with a history of well-controlled asthma since the age of 20, who was initially consulted for dyspnea stage III of the Modified Medical Research Council score (mMRC). Chest X-ray showed moderate bilateral pleural effusion. The pleural biopsy concluded with a pleural metastasis of breast carcinoma.

## Introduction

Malignant pleural effusions are common among patients with neoplastic diseases [1]. They also represent 42%-77% of exudative effusions [1]. And among all the malignancies associated with this entity, breast cancer is the primary cause in more than half the cases in women.

Lobular breast carcinomas are not excluded as they represent the second most frequent histological form of breast carcinomas (5%-15%) after infiltrating duct carcinomas [2]. The spread patterns of metastatic lobular and ductal carcinoma of the breast are different. Lobular carcinoma frequently metastasizes to the peritoneum, gastrointestinal tract, bones, and ovaries with a lower incidence of spreading to the lungs or pleura [3]. Pleural effusions in these malignancies usually appear at an advanced stage of the disease. However, they can be the revealing manifestation as proven by our case study.

Management decisions should be based on the individual patient and the biological characteristics of the tumor. However, lobular carcinoma has been reported to be less responsive to chemotherapy [4].

## Case presentation

We report the case of a 61-year-old woman, with a history of well-controlled asthma diagnosed at the age of 20 and treated with inhaled corticosteroids in combination with long-acting beta2-agonists, who consulted for stage III of Modified Medical Research Council score (mMRC) dyspnea, accompanied by wheezing and pleuritic chest pain for two months. The physical examination noted a weight loss of 13 kg in two months.

On presentation, her blood pressure was 120/70 mm Hg, pulse was 95/min and regular, respiratory rate was 25 breaths/min, and oxygen saturation measured by pulse oximetry was 90% on room air. The respiratory examination indicated decreased breath sounds in the bilateral middle lung fields with dullness on percussion.

Breast examination revealed a mobile lump of 1 cm in the upper outer quadrant of the left breast with no palpable axillary nodes. There was a retraction of the nipple; the nodule, which was mobile, had a hardened consistency and did not adhere to deep structures.

**Figure 1:**
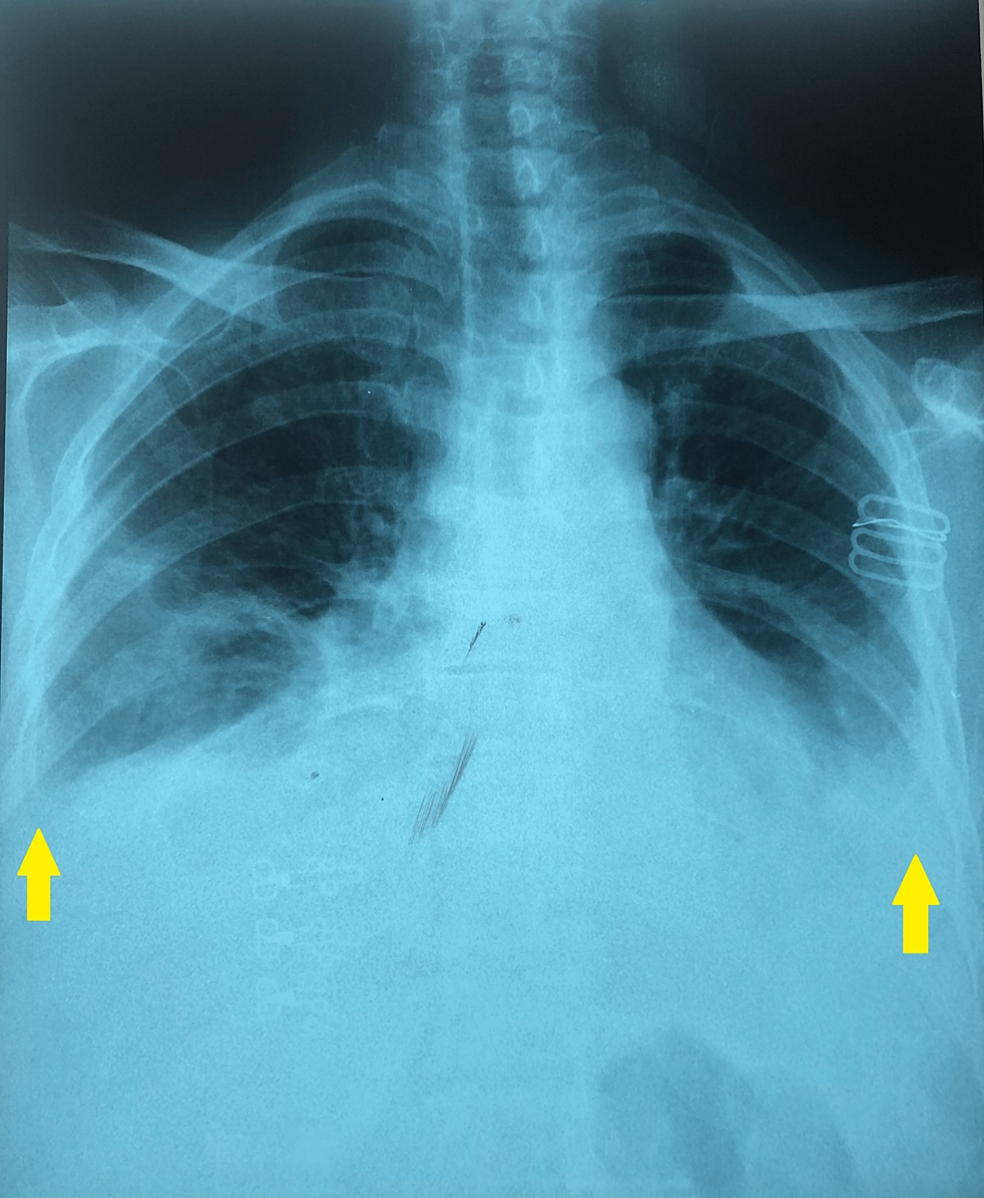
Chest X-ray revealed moderate bilateral pleural effusion (yellow arrows).

She had no lymphadenopathy. The remainder of the examination was normal. Chest X-ray showed a moderate bilateral pleural effusion (Figure 1). The chest CT scan also confirmed the bilateral pleural effusion.

Analysis of the right-side pleural fluid obtained by thoracentesis indicated an exudate with elevated fluid protein, whereas serum protein, fluid lactate dehydrogenase, and serum lactate dehydrogenase were within the normal range (Table 1). No acid-fast bacilli were detected in the pleural fluid and cytology was negative for malignant cells. A percutaneous biopsy of the right-side pleura was performed, and the diagnosis was pleural metastasis of breast carcinoma.

**Table 1:** Laboratory results of the right-side pleural fluid obtained by thoracentesis.

Laboratory test	Value	Reference range
Fluid protein (g/L)	45	<20
Serum protein (g/L)	70	60-80
Fluid lactate dehydrogenase (U/L)	145	<200
Serum lactate dehydrogenase (U/L)	202	140-280

**Figure 2:**
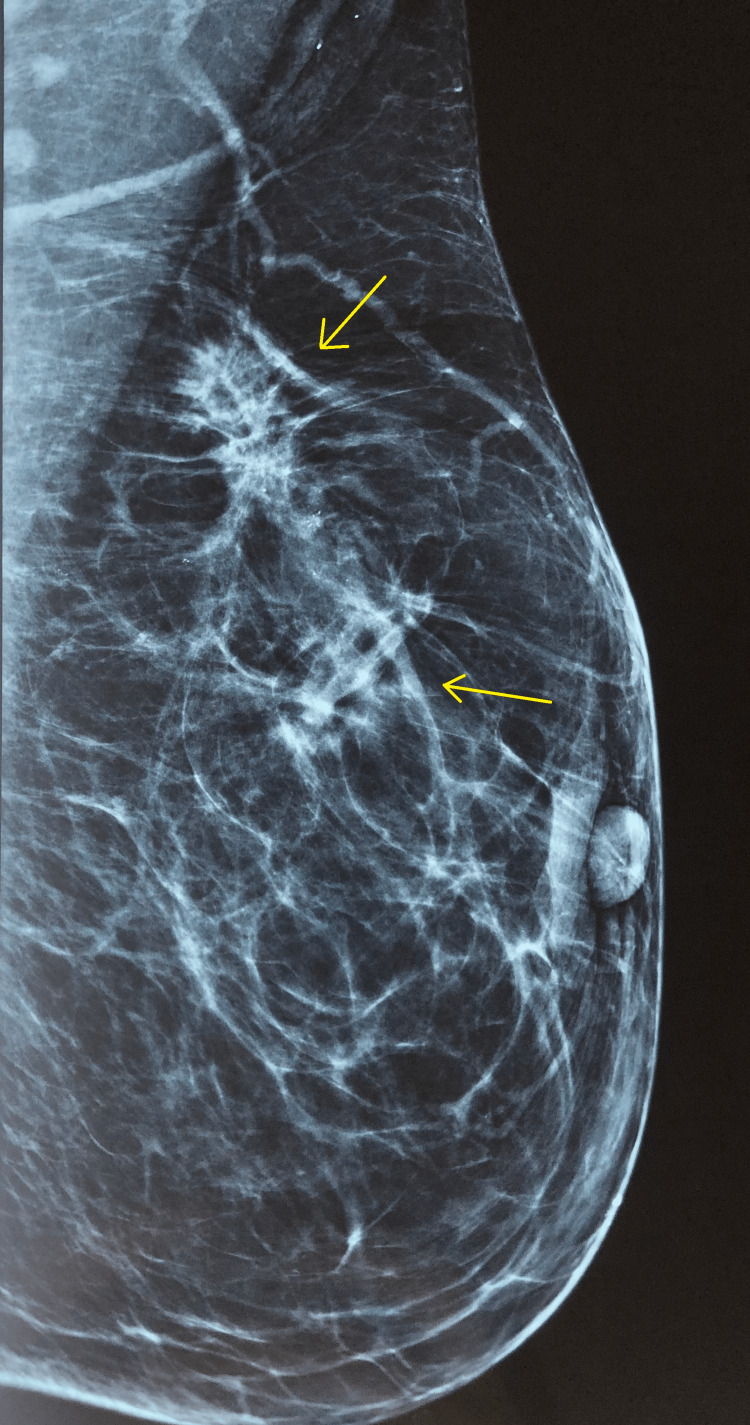
Left mammography revealed a nodule of irregular shape measuring 24 × 13 mm in the upper outer quadrant of the left breast and another nodule measuring 12 × 10 mm at the junction of the upper quadrants (yellow arrows).

Mammography and ultrasound revealed a nodule of the irregular shape measuring 24 × 13 mm in the upper outer quadrant of the left breast and another nodule measuring 12 × 10 mm at the junction of the upper quadrants of the same breast. The final Breast Imaging Reporting and Data System (BI-RADS) category was 5, highly suggestive of malignancy (Figure 2). The contralateral breast results were normal. We also add that these were the first mammogram and ultrasounds ever done for the patient.

**Figure 3:**
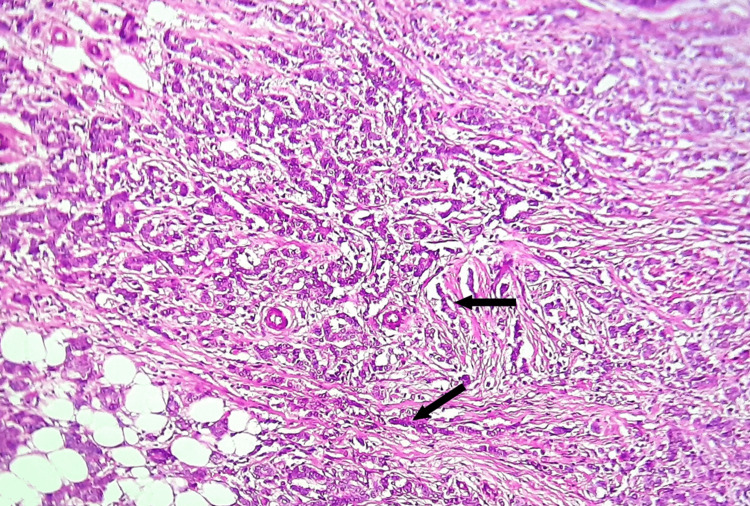
Histological section showing tumor proliferation arranged in single files (arrow), cords, single cells, and rare glands. The tumor cells were discohesive, small, monomorphic and lacked marked atypia.

A core needle biopsy was performed, and the diagnosis was a grade II invasive lobular carcinoma with progesterone and estrogen receptors both being positive (Figure 3). A thorax, abdomen, and pelvis CT scan was performed in order to determine the metastatic workup of the tumor that detected bilateral pleural effusion, two nodules of spiculated contours in the left breast, and multiple lesions in the skull and the peripheral skeleton bones. The staging of the tumor was completed by a brain and cervical CT scan which was without abnormalities. 

The patient was then referred to another facility for further treatment.

## Discussion

Breast cancer is the second leading cause of death after lung cancer in women [5], as nearly a quarter of breast cancers develop metastasis during the course of tumor progression, and about 5% are metastatic at the time of the first cancer diagnosis [6]. Infiltrating lobular carcinoma (ILC), which represents 5% to 15% of all breast cancers, tends to stay clinically silent and escapes detection on a mammogram or physical examination until the disease is detected at advanced stages [7]. We report a case of breast lobular carcinoma with bilateral pleural effusion as the first presenting sign.

In clinical breast examinations, vague findings such as thickening or induration are found. In particular, clinical examination and early diagnosis of ILC are very challenging in patients with dense breast tissue or fibrocystic mastopathy. A high incidence of contralateral tumors in patients with ILC has been claimed [8]. In our case, the breast lesion was unilateral.

In metastatic disease, ILC presents with some distinctive patterns of metastatic dissemination compared to other histological breast cancer types. A higher incidence of spreading to the peritoneum, gastrointestinal tract, bone, and gynecologic tract was observed [9].

Pleural metastasis is exceptional in ILC and appears at an advanced stage of the disease. It can rarely reveal a primary tumor. In a study by Cellerin et al., which focused on 209 malignant pleural effusions, ovarian and lung tumors (27%) were the most frequent primary site revealed by pleural effusions in women, followed by mesothelioma (19.4%) [10]. In only two patients, breast cancer was revealed by a pleural effusion. However, when the pleural effusion is metastatic to a known cancer, the breast was the primary site found in half the cases (50%) followed by lung cancer (14.5%) and lymphoma (10.5%) [10].

The study by Monte et al. of 126 malignant pleural effusions showed that mammary carcinoma did not present initially with effusion in any case, although it was the most common malignant tumor found in cases of pleural effusions [11].

In a study of 105 patients with breast cancer who had a pleural effusion as a direct consequence of metastatic disease, Fentiman et al. noted that 48% of these tumors metastasized ipsilaterally, 42% contralaterally, and 10% of tumors produced bilateral pleural effusions, as was the case with our patient [12].

Patients most commonly present with dyspnea, initially on exertion, predominantly dry cough, and pleuritic chest pain [6]. In a study by Yahiaoui et al, out of 158 patients, the diagnosis of malignant pleural effusion was confirmed by cytology in 71 cases (6%), by closed percutaneous needle biopsy in 55 cases (2%), and by thoracoscopy in 10 cases (4%) [13]. In a study of 170 malignant effusions, Aidou reported that needle biopsy was 75% contributory [14]. In our case, the diagnosis was made by blind pleural biopsy.

Patients with lobular carcinoma are more often treated with hormone therapy than patients with ductal carcinoma because of the higher frequency of hormone receptor-positive cells. Neoadjuvant chemotherapy is less recommended given the poor response unless the tumor is inoperable from the start [15].

For the management of malignant pleural effusion, although chemotherapy or hormone therapy can in some cases achieve at least temporary resolution of the effusion, it will sooner or later recur. Among the techniques available, isolated pleural drainage is ineffective [16,17]. External radiotherapy is also ineffective on the pleura. Pleurectomy has a cost-effectiveness of close to 100%, but it is associated with significant morbidity and mortality and is nowadays abandoned in this indication [16]. Therefore, pleurodesis is the local treatment of choice for recurrent pleurisy requiring repeated evacuation [18].

For our patient, the decision was to start hormone therapy and chemotherapy; she also benefited from a thoracentesis on both sides without recurrence after four months of evolution.

## Conclusions

The association between breast cancer and pleural effusion is frequent. However, pleural effusion may reveal cancer in rare situations, indicating an advanced stage of the disease. The treatment of pleural effusion, if the indication arises, is essentially based on pleurodesis allowing the achievement of definitive pleural symphysis as well as an improvement in the quality of life in patients.
